# Genetic Alterations in Chromatin Regulatory Genes in Upper Tract Urothelial Carcinoma and Urothelial Bladder Cancer

**DOI:** 10.1002/cam4.70398

**Published:** 2024-11-08

**Authors:** Shuo Wang, Xuzhi Yan, Weihua Lan, Yapeng Wang, Ze Wang, Dali Tong, Yao Zhang, Qiang Ran, Haoyang Li, Junhao Jin, Haiyang Xiao, Jing Xu, Qian Yan, Dianzheng Zhang, Qiang Ma, Hualiang Xiao, Jun Qin, Luofu Wang, Jun Jiang, Qiuli Liu

**Affiliations:** ^1^ Department of Urology, Daping Hospital Army Medical University Chongqing People's Republic of China; ^2^ School of Basic Medical Science Army Medical University Chongqing People's Republic of China; ^3^ Department of Bio‐Medical Sciences Philadelphia College of Osteopathic Medicine Philadelphia Pennsylvania USA; ^4^ Department of Pathology, Daping Hospital Army Medical University Chongqing People's Republic of China; ^5^ CAS Key Laboratory of Tissue Microenvironment and Tumor, CAS Center for Excellence in Molecular Cell Science, Shanghai Institute of Nutrition and Health Sciences, Chinese Academy of Sciences University of Chinese Academy of Sciences Shanghai People's Republic of China

**Keywords:** chromatin regulatory genes, clonal relatedness, upper tract urothelial carcinoma, urothelial carcinoma of bladder

## Abstract

**Purpose:**

Upper tract urothelial carcinoma (UTUC) and urothelial carcinoma of the bladder (UCB) share histomorphological and therapeutic features but distinct epidemiologic and clinicopathologic characteristics. We examined alterations of chromatin regulatory genes in molecular subtypes, clonal relatedness, and T‐cell receptor (TCR) diversity in UTUC and UCB.

**Materials and Methods:**

Targeted next‐generation sequencing or whole‐exome DNA sequencing and TCR sequencing were conducted with 34 UTUC and 49 UCB specimens from 63 patients. Tumors were subtyped based on the expression of CK5 and GATA3. Results of tissue microarray of 78 muscle‐invasive bladder cancer (MIBC) samples were used as prognostic factors of different subtypes of MIBC.

**Results:**

Chromatin regulatory genes were frequently mutated in both UTUC and UCB. Rapid relapse and progression of non‐MIBC are correlated with alterations of *KMT2C* and *EP300*. Frequency of alterations in chromatin regulatory genes is higher in UTUC patients with SBS22 and SBS2 signatures and lower in UCB patients with SBS2 and SBS6 signatures. GATA3 and CK5 double‐positive patients with higher frequencies of *SMARCA4*, *ARID1A*, and *EP300* mutations have better prognoses than patients with basal subtypes. Although UTUC and UCB in the same patient can be either clonally related or developed independently, mutated genes in chromatin pathway were enriched in the related clones. Compared to UTUC, UCB had more deleterious mutations in DNA damage repair (DDR) genes, higher levels of tumor mutation burden (TMB) and copy number variations (CNVs), as well as higher TCR clonality and lower TCR diversity.

**Conclusions:**

Since genetic alterations of the chromatin pathway genes are important in both UTUC and UCB, they could serve as potential biomarkers for predicting disease progression and therapeutic targets. Differences in mutation frequencies of DDR pathway, TMB, CNV, and TCR might be the contributing factors for the distinct responses to immune checkpoint inhibitor (ICI) between UTUC and UCB.

## 1 Introduction

Urothelial carcinoma (UC) is the 12th most common malignancy worldwide [1, 2]. UC can be divided into upper tract urothelial carcinoma (UTUC) or urothelial carcinoma of bladder (UCB). Although UTUC and UCB share histomorphological and therapeutic similarities, they differ epidemiologically and clinicopathologically [3]. UCB accounts for 90%–95% of UC, and UTUC accounts for the remainder [4]. The incidence of UCB is estimated to be 9.5/100,000 in men and 2.4/100,000 in women, while the incidence of UTUC is 2/100,000 [5, 6]. About two‐thirds of UTUC are invasive, and only a quarter of UCB is invasive [3, 7]. The 5‐year cancer‐specific survival for invasive UCB is around 65% versus 50% for invasive UTUC [3, 7]. The tumorigenesis and progression of UCB are closely related to tobacco exposure, while UTUC is more common than UCB in patients with Lynch syndrome [8]. Moreover, UTUC and UCB patients have different response rates to immunotherapy [9]. However, the contributive factors for the intrinsic differences between UTUC and UBC are largely unknown. In this study, we tried to determine whether differences in the spectrum of genetic alterations could explain above‐mentioned clinical differences between UTUC and UCB.

Epigenetic dysregulation and chromatin remodeling abnormalities are widely linked to cancer initiation, progression, and resistance to therapies [10, 11, 12]. The frequency of mutations in chromatin regulatory genes is consistently high in UC, along with mutations in PI3K/AKT and cell cycle and TP53/RB1 signaling pathway genes, [13] and aberrations in chromatin regulation are a hallmark of bladder cancer [14]. Audenet et al. showed a high frequency of mutations in chromatin regulatory genes in UTUC [15]. Genomic profiling has improved UCB diagnosis, prognosis, and therapeutic choices [16, 17, 18]. Comparatively, genomic profiling of UTUC is less advanced [19]. Previous studies have revealed some biological differences between UTUC and UCB [15, 20]. For example, microsatellite instability and promoter methylation are more common in UTUC [21, 22]. *TP53*, *RB1*, and *ERBB2* were less frequently altered in UTUC compared with UCB [15]. Considering important roles of chromatin regulatory genes in both UTUC and UCB, we wondered if there are different frequencies of genetic alterations in these genes that might contribute to the distinct clinical characteristics.

UTUC and UCB often co‐occur in individual patients. About 17% of UTUC patients are found with the concomitant UCB at the time of diagnosis. Following radical nephroureterectomy for nonmetastatic UTUC, about 22% to 47% of the patients were diagnosed with UCB within 2 years, whereas only 2.6% of nonmuscle‐invasive UCB have a subsequent UTUC [23]. To better understand tumor heterogeneity and clonal relatedness, we examined the genetic alterations in UTUC and UCB, especially for those raised from the same patients.

## 2 Materials and Methods

### 2.1 Patients and Samples

UTUC (*n* = 34) and UCB samples (*n* = 49) from 63 patients, including 20 patients with both UTUC and UCB, were collected between March 2018 and January 2022 with the approval of the Research Ethics Committee of Daping Hospital, Army Medical University (No: 201896), and written informed consent from each patient. The clinical information of these patients is shown in Table S1. All procedures involving human participants were carried out in accordance with the ethical standards of the institutional research committee and with the 1964 Helsinki Declaration and its later amendments or comparable ethical standards. The pathological diagnosis was performed by two experienced pathologists (Dr. Qiang Ma and Hualiang Xiao, Department of Pathology, Daping Hospital) according to Union Internationa le Contrele Cancer (8th edition). Tissue microarray with another cohort of 78 muscle‐invasive bladder cancer (MIBC) patients was used for molecular typing of bladder cancer, and the clinical information of these patients is shown in Table S2.

### 2.2 Library Preparation and Next‐Generation Sequencing

Sequencing was conducted commercially by targeting 87 (*n* = 3), 891 (*n* = 11), and 1054 (*n* = 29) cancer‐related genes or whole exons (*n* = 40). DNA was extracted from formalin‐fixed paraffin‐embedded sections of UTUC/UCB tumors, fresh tissue specimens, and blood samples using TIANamp Genomic DNA kit (DP304, Tiangen Biotech, Beijing, China) as described previously [24, 25]. Germline DNAs from white blood cells were used as a normal control for somatic mutations discovery. Whole‐exon sequencing (WES) libraries were generated using NEB Next Ultra II DNA Kits (NEB, E7654). DNA for the target region library was enriched using custom probe sets from IDT (Integrated DNA Technologies) and was sequenced using the MIG T7 sequencer (BGI, Shenzhen, China). Targeted sequencing libraries were generated using KAPA Library Preparation Kit (KAPA, Biosystems, Wilmington, MA, USA). The SeqCap EZ Library System (Roche NimbleGen, Madison, WI, USA) was used to enrich the target regions for four panels comprising 87, 891, and 1054 cancer‐related genes (Table S3). The sequence reads were aligned to the human reference genome (GRCh37/hg19) using the Burrows Wheel Aligner software (version 0.7.10). All tumor tissues and control samples (adjacent tissues or blood samples) were under the same sequencing platform. Somatic single nucleotide variants (SNVs) and small fragments insertions and deletions (indels) were analyzed using MuTect (1.1.4). GATK software (version 4.0) was used to analyze somatic copy number variations (CNVs). For the mutational signature analysis, only nonsynonymous single base substitutions (SBSs) were extracted by nonnegative matrix factorization and mapped to the 96 mutational signatures from the Catalog of Somatic Mutation in Cancer (COSMIC) database (https://cancer.sanger.ac.uk/signatures/sbs/) with R package sigminer (v2.2.7) [26, 27].

### 2.3 DNA Damage Repair (DDR) Gene Mutation Analysis

Mutations in 34 DDR genes that are associated with ICI response (Table S4) [28] were analyzed using the following criteria [29]: (1) all nonsense mutations, frameshift mutations, and splice site mutation; and (2) missense mutation if pathogenic in COSMIC (https://cancer.sanger.ac.uk/cosmic) or ClinVar (https://www.ncbi.nlm.nih.gov/clinvar/) or with a Polyphen‐2 score [30] ≥ 0.95.

### 2.4 Immunohistochemical Staining

Paraffin‐embedded tissue sections of 4 μm were baked at 60° C for 6 h, deparaffinized with xylene, and rehydrated in graded ethanol and 3% hydrogen peroxide to block the endogenous peroxidase activity. The sections were submerged in citrate or EDTA buffer and microwaved for antigen retrieval. Goat serum (ZSGB‐BIO, China) was used to block nonspecific background signals, and the sections were incubated at 4°C with specific antibodies against CK5 (ZA‐0518, ZSJQ‐BIO), GATA3 (ZA‐0661, ZSJQ‐BIO), Ki67 (ZM‐0166, ZSJQ‐BIO), CD3 (ab5690, Abcam), CD4 (ZM‐0418, ZSJQ‐BIO), CD8 (ZA‐0508, ZSJQ‐BIO), and PD‐L1 (SP142, Roche) followed by the secondary antibody (HRT‐Polymer Anti‐Rabbit/Mouse, KIT‐5030, MaxVision, China). T‐cell density and PD‐L1 were analyzed by the methods described previously [31]. Microscopy images of the slides were captured by NIS‐Elements F3.2 and evaluated by two urological pathologists (Dr Qiang Ma and Hualiang Xiao).

### 2.5 Clonality Assessment and Clonal Evolution Analysis

Clonality assessment and clonal evolution analysis were conducted using the WES results from 20 patients with both UTUC and UCB. Firstly, we filtered the mutation information read by Maftool using a variant allele frequency (VAF) > 0.1. Then, we determined the clonal relatedness of the paired tumors in the same patient using all SNPs, CNVs, and indels, including synonymous mutations by the R package Clonality (v1.44.0) [32], as described previously [33]. Pyclone (0.13.1) [34] was used to infer clone and subclone clusters, and CloEvol (0.99.11) [35] was used to visualize the data and construct the phylogenetic trees.

### 2.6 T‐cell Receptor (TCR) β‐Chain Sequencing and Data Analysis

TCR β‐chain sequencing was conducted using the 20 paired UTUC and UCB samples. Tissue DNA was extracted as described above. The highly variable complementary‐determining region 3 (CDR3) of TCR β chains was amplified for all possible V(D)J combinations with 32 forward primers of V genes and 13 reverse primers of J genes by QIAGEN Multiplex PCR Kit (Cat. No: 206145) [25]. The DNA libraries were measured by Qubit 2.0 Fluorometer (Q32866, Thermo Fisher Scientific) with Quanti‐IT dsDNA HS Assay Kit (Q33120, Thermo Fisher Scientific) and Agilent Bioanalyzer 2100 (G2939B, Agilent). Samples with a DNA library concentration ≥ 20 ng/uL were then performed by high‐throughput sequencing on the MGI2000 platform. MIXCR software (v3.0.3) was used to compare the sequences with the ImMunoGeneTics (IMGT) database to determine the V, D, and J gene sequences, identify the CDR3 sequence of the TCR, and then translate the nucleotide sequence [36]. VDJtools (v1.2.1) was used for statistical analysis of T‐cell diversity (calculated using the Shannon index) and T‐cell clonality (calculated using clonality).

### 2.7 Statistical Analysis

We used chi‐squared and Fisher's exact test to analyze the differences in the clinical data and the alterations between UTUC and UCB cohorts. The independent samples *t*‐test (two‐sample *t*‐test) was used for enumeration data. Nonparametric tests were used when normal distribution and homogeneity of variance were not satisfied. Cox regression analysis was used to calculate the association between mutations and clinical progression. Survival outcomes for molecular subtypes were analyzed using the log‐rank test and Kaplan–Meier curves. The paired *t*‐test or Wilcoxon matched‐pairs signed‐rank test was used to compare the TCR diversity of paired samples. Statistical analyses were performed using GraphPad Prism 8. *p* < 0.05 was considered statistically significant.

## 3 Results

### 3.1 Chromatin Regulatory Genes are Frequently Mutated in UTUC and UCB

**FIGURE 1:**
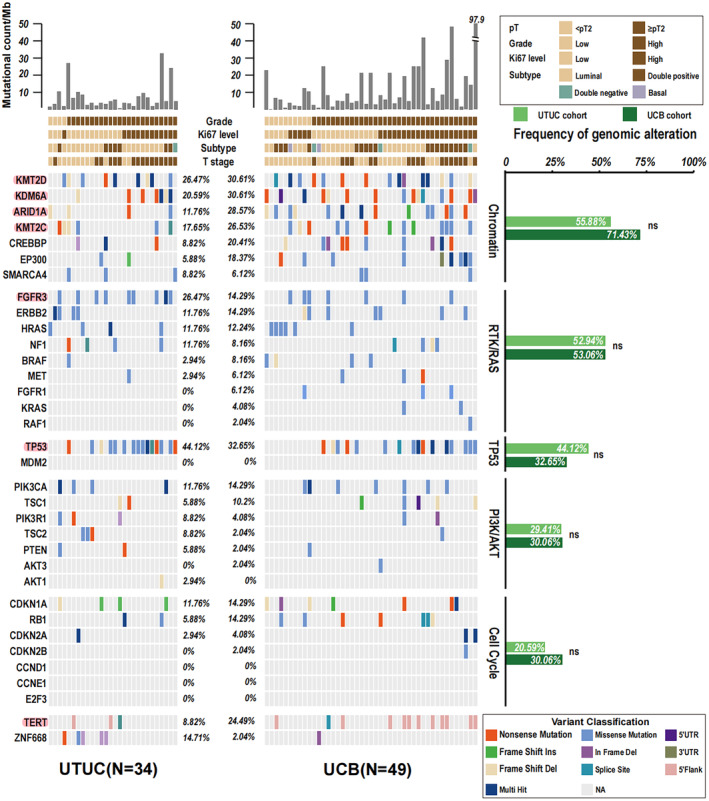
Genetic alterations of UTUC and UCB stratified by clinical state and molecular pathways. The top mutated genes are highlighted by red boxes. Mutation information for 6 UTUC and 4 UCB samples is omitted as no mutations were detected in the genes shown.

The characteristics of the 63 patients are summarized in Table S1. DNA sequencing of 34 UTUC and 49 UCB specimens revealed that the most frequently mutated genes in UTUC were *TP53* (44%), *FGFR3* (26%), *KMT2D* (26%), *KDM6A* (21%), *KMT2C* (18%), and *ARID1A* (12%). Of note, FGFR3 (40%), KMT2D (37%), KDM6A (32%), TP53 (26%), and ARID1A (23%) also ranked top 5 in TCGA UTUC cohort [15]. Notably, TP53 mutations were found with higher frequency in our UTUC cohort (*p* = 0.033, Figure S1A). The most frequently mutated genes in UCB were *TP53* (33%), *KDM6A* (31%), *KMT2D* (31%), *ARID1A* (29%), *KMT2C* (27%), and *TERT* (24%) (Figure 1). In TCGA UCB cohort, *TP53* (57%), *KDM6A* (41%), *KMT2D* (30%), *ARID1A* (28%), and *FGFR3* (19%) ranked top 7 [37]. Of note, TP53 mutations were found with lower frequency in our UCB cohort (*p* = 0.005, Figure S1B). All the mutations of *TERT* were at its promoter region. As described previously [38], these genes are mainly involved in five pathways/processes including chromatin, RTK/RAS, TP53/MDM2, PI3K/AKT, and the cell cycle. Of note, *KDM6A*, *KMT2D*, *KMT2C*, and *ARID1A* are all chromatin regulators suggesting that changes in the regulation of chromatin are important in both UTUC and UCB. Further mutational analysis found that loss‐of‐function mutations frameshifts, nonsense substitutions, or alternative splicing have higher frequencies (Figure S1C,D). Although the mutation frequencies of these genes and the five signaling pathways were similar in UTUC and UCB (Figure 1 and Figure S1E), mutation frequencies for chromatin regulatory genes were relatively higher in UCB than in UTUC (71% vs. 56%, *p* = 0.22), especially *ARID1A* (29% vs. 12%, *p* = 0.068) (Figure S1E). For the nonchromatin regulatory genes, the mutation frequency of *TERT* was relatively higher in UCB than in UTUC (24% vs. 9%, *p* = 0.086) (Figure S1E), but the mutation frequencies of *ZNF668* (15% vs. 2%, *p* = 0.04) (Figure S1F) and *FGFR3* (26% vs. 14%, *p* = 0.17) (Figure S1E) were higher in UTUC than in UCB.

### 3.2 Mutations in Chromatin Regulatory Genes and Progression of UTUC and UCB

**FIGURE 2:**
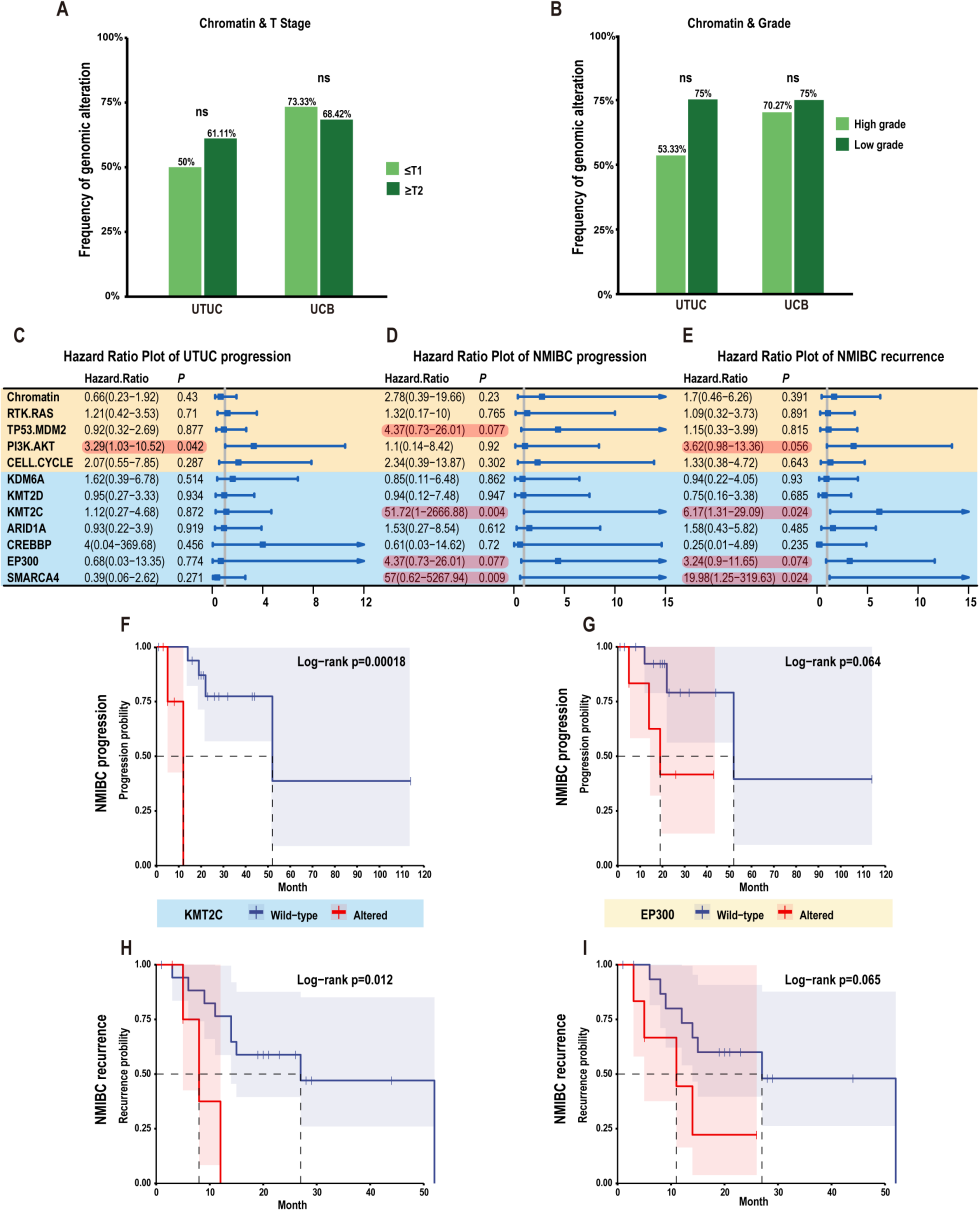
Genetic alterations associated with tumor stage, grade, and prognosis. (A and B) The mutation frequencies of chromatin pathway genes in different tumor stages (A) and grades (B). (C–E) The relationship between genetic alterations of indicated pathways or genes and UTUC progression (C), and the progression (D) and recurrence (E) of nonmuscle‐invasive bladder cancer (NMIBC). (F–I) Kaplan–Meier analysis shows the associations of *KMT2C* (F and H) and *EP300* (G and I) mutations with the progression and recurrence of NMIBC.

In both UTUC and UCB, the mutation frequencies of genes involved in chromatin signaling pathways were similar for various tumor stages/grades (Figure 2A,B, Table S5). Mutations in chromatin signaling pathways were not associated with UTUC progression or recurrence nor the progression of nonmuscle‐invasive bladder cancer (NMIBC) to MIBC (Figure 2C–E). However, patients with alterations in chromatin regulators *KMT2C* (HR 51.72, *p* = 0.004) and *EP300* (HR 4.37, *p* = 0.009) progressed more rapidly to MIBC (Figure 2D,F–G). Mutations in TP53/MDM2 pathway genes were more frequent in both high‐stage (≥ T2) UTUC (*p* = 0.007) and high‐grade UCB (*p* = 0.005) (Figure S2A,B). Consistently, we saw increased progression from NMIBC to MIBC in patients with alterations in TP53/MDM2 pathway genes (HR 4.37, *p* = 0.08). The mutation frequencies in RTK/RAS pathway genes were higher in low‐grade UCB (*p* = 0.02) but not UTUC (Figure S2C). Mutations in PI3K/AKT pathway genes were associated with greater UTUC progression (HR 3.29, *p* = 0.04) and NMIBC recurrence (HR 3.62, *p* = 0.06). Furthermore, the risk of NMIBC recurrence increased by mutations in *KMT2C* (HR 6.17, *p* = 0.02) and *EP300* (HR 3.24, *p* = 0.07) (Figure 2E,H–I). Finally, the number of Ki‐67‐positive cells in NMIBC is associated with both NMIBC progression and recurrence (Figure S2D–F).

### 3.3 Mutations in Chromatin Regulatory Genes and Mutational Signatures

**FIGURE 3:**
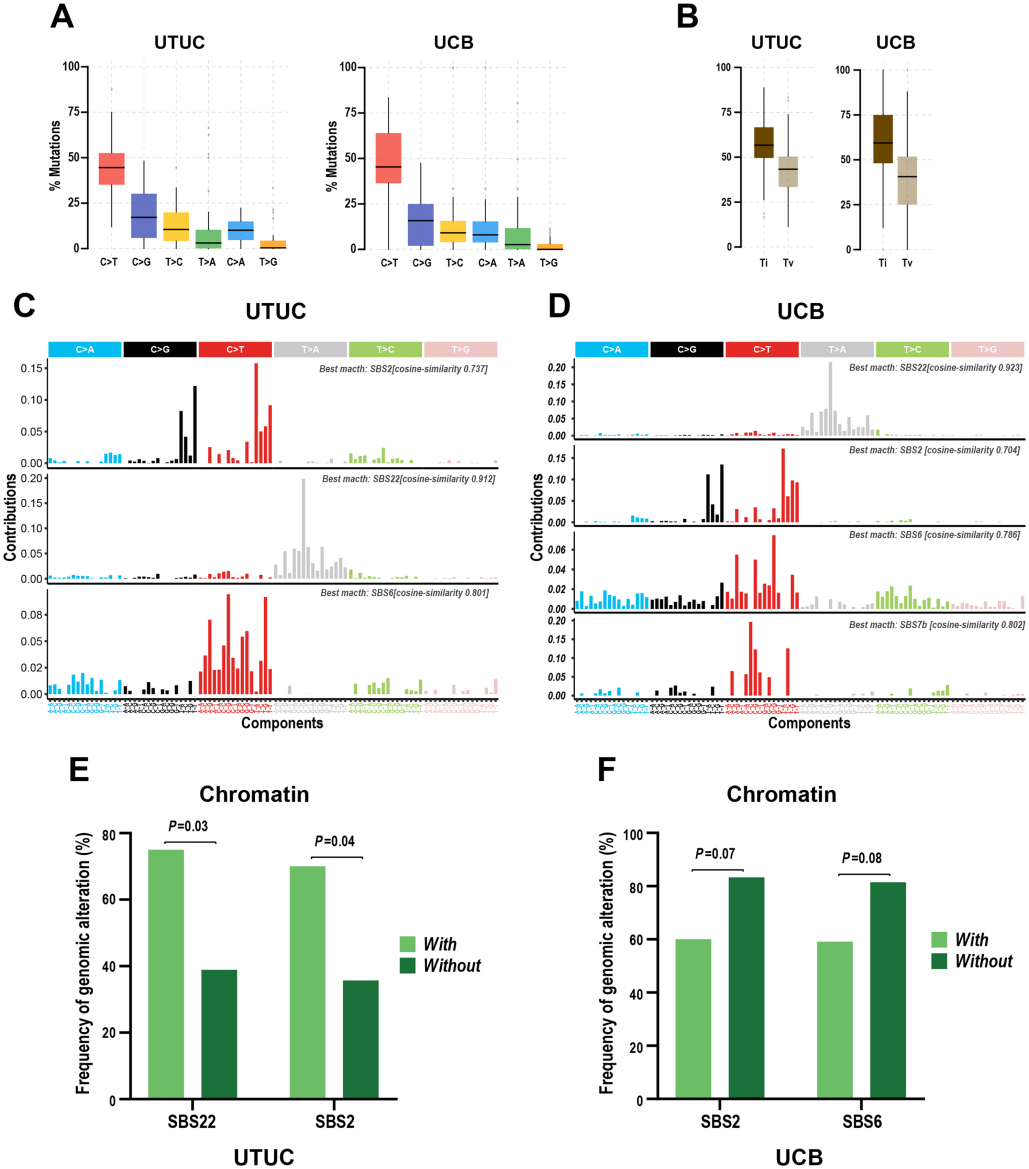
Dominant mutational signatures of UTUC and UCB. (A) Single base substitution patterns in UTUC and UCB cohorts. (B) Proportion of transitions (Ti) or transversion (Tv) in UTUC and UCB cohorts. (C) Mutational signatures in UTUC cohort. (D) Mutational signatures in UCB cohort. (E and F) Relationship between mutational signatures (shown with and without) and genetic alterations in chromatin pathway genes in UTUC (E) and UCB (F).

We identified mutational signatures specific to UTUC and UCB. In both UTUC and UCB, C to T substitution (Figure 3A, Figure S3A,B) and transitions (A↔G; C↔T) (Figure 3B) were the predominant mutations. Nonnegative matrix factorization using the COSMIC database revealed signature 22 (exposure to aristolochic acid), signature 6 (defective DNA mismatch repair), and signature 2 (AID/APOBEC mutagenesis) in both UTUC and UCB (Figure 3C,D). However, the SBS7b mutation (exposure to ultraviolet light) is specific to UCB (Figure 3D). Higher frequencies of mutations in chromatin regulatory genes in UTUC patients were accompanied by SBS22 (*p* = 0.03) and SBS2 (*p* = 0.04) signatures (Figure 3E). In UTUC patients, the SBS22 signature is associated with higher frequencies of mutations in genes in the RTK/RAS pathway (Figure S3C). In UCB patients, SBS2 and SBS6 signatures are associated with lower frequencies of mutations in chromatin regulatory genes (Figure 3F), and the SBS6 signature is associated with lower frequencies of mutations in genes in the PI3K/AKT pathway (Figure S3D).

### 3.4 Mutations in Chromatin Regulatory Genes and Molecular Subtype

**FIGURE 4:**
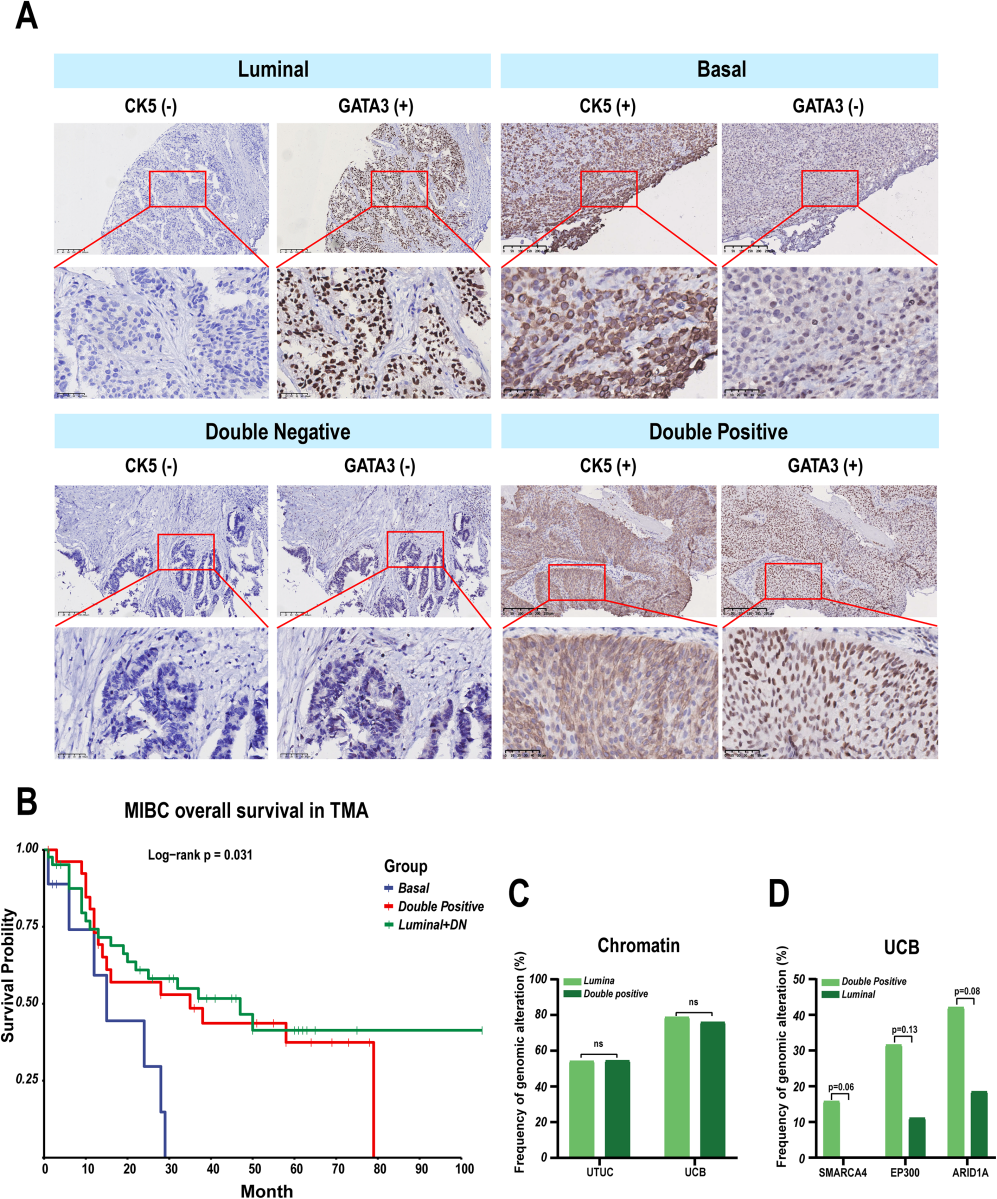
Molecular subtype determined by immunohistochemical staining for CK5 and GATA3. (A) Representative immunohistochemical staining from samples with luminal (CK5^−^/GATA3^+^), basal (CK5^+^/GATA3^−^), double‐negative (CK5^−^/GATA3^−^), and double‐positive (CK5^+^/GATA3^+^) subtypes. (B) Overall survival analysis of another MIBC cohort with diverse subtypes in our tissue microarray. Double negative, DN. (C) Mutation frequencies of chromatin pathway genes in patients with indicated subtypes. (D) Genetic alterations of *EP300* in MIBC patients with indicated subtypes.

The levels of CK5 and GATA3 can be used for subtyping luminal versus basal MIBC with ~90% accuracy [39, 40]. Based on these criteria [39], 23 UTUC and 25 UCB luminal subtypes are either GATA3^+^/CK5^−^ or double negative. Three basal subtype UCBs are CK5^+^/GATA3^−^ (Figure 4A). A subset of GATA3^+^/CK5^+^ UTUC (11/34, 32%) and UCB (19/49, 39%) (Figure 4A and Figure S4A) has not been taken notice of previously [39, 40]. Expression of GATA3 and CK5 in our tissue microarrays suggests that overall survival is similar for patients with GATA3^+^/CK5^+^ MIBC and luminal MIBC. The overall survival for patients with basal MIBC is lower than those with either luminal MIBC (HR 2.489, *p* = 0.04) or GATA3^+^/CK5^+^ MIBC (HR 2.405, *p* = 0.04) (Figure 4B and Figure S4B). In addition, the mutation frequency for chromatin regulatory genes in GATA3^+^/CK5^+^ MIBC is similar to the luminal subtype in both UTUC and UCB cohorts (Figure 4C). However, alterations in *SMARCA4* have been found in GATA3^+^/CK5^+^ UCB but not luminal UCB (Figure 4D), and alterations of *ARID1A* and *EP300* are more frequent in GATA3^+^/CK5^+^ UCB than the luminal subtype (Figure 4D).

### 3.5 The Chromatin Pathway in Clonal Relatedness of UTUC and UCB

**FIGURE 5:**
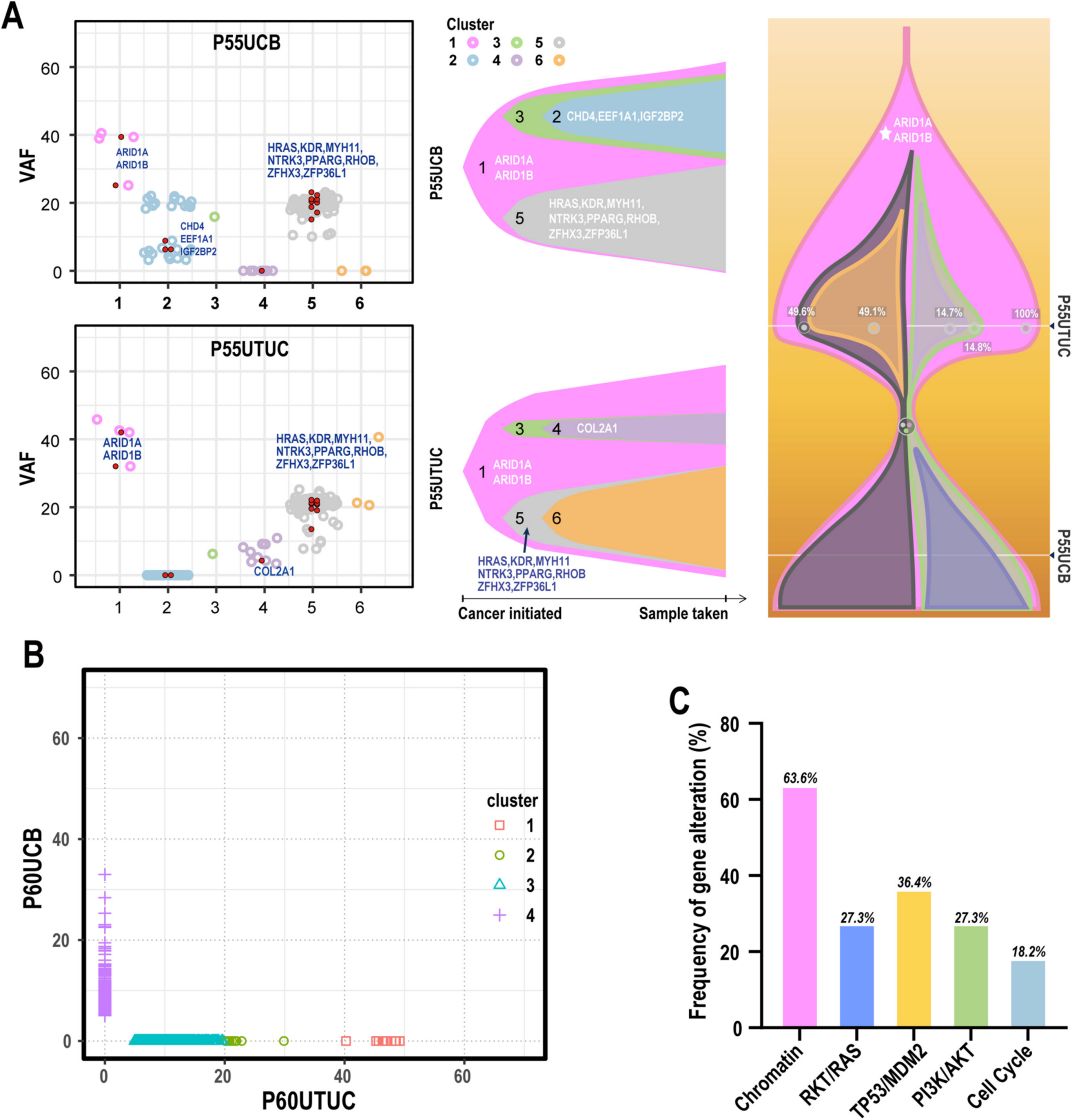
Tumor evolution analysis. (A) The clonal evolution of UTUC and UCB from the representative patient with the same clonal origin. The left panel shows the clones and subclones. Variant allele frequency, VAF. The Sankey plot in the middle panel shows the evolutionary models. The right Sankey plot shows the deduced clonal evolution of paired UTUC and UCB. (B) Representative scatterplot showing completely different subclones in a representative patient with different clonal origin. (C) The pathways in which the genetic alterations of the shared clones of UTUC and UCB were enriched.

To understand the roles of different pathways in clonal relatedness between UTUC and UCB, WES was conducted for paired UTUC and UCB from 20 patients [33]. Tumors in 11 of the 20 patients (55%) shared a clonal origin (*p* < 0.0001) (Figure S5 and Table S6). Of these 11 patients, four had both UTUC and UCB at their diagnosis, four had UCB and developed UTUC, and three had UTUC and developed UCB, suggesting that clonal relatedness is independent of the sequence of the cancer occurrence. Phylogenetic trees indicated that the representative patient who had UTUC first and later developed UCB share common ancestral *ARID1A* and *ARID1B* mutations, whereas alterations of multiple driver genes, including *HRAS*, *KDR*, *MYH11*, *NTRK3*, *PPARG*, *RHOB*, *ZFHX3*, and *ZFP36L1*, occur later (Figure 5A). Oppositely, the UTUC and UCB tumors from the rest of the nine patients have no clonal relatedness just as shown in Figure 5B. Importantly, the shared mutant genes had the highest frequency of mutations (63.6%) enriched in chromatin pathway (Figure 5C), suggesting that this pathway is important in clonal relatedness.

### 3.6 Pathway Mutations and Tumor Sensitivity to Immune Checkpoint inhibitor

**FIGURE 6:**
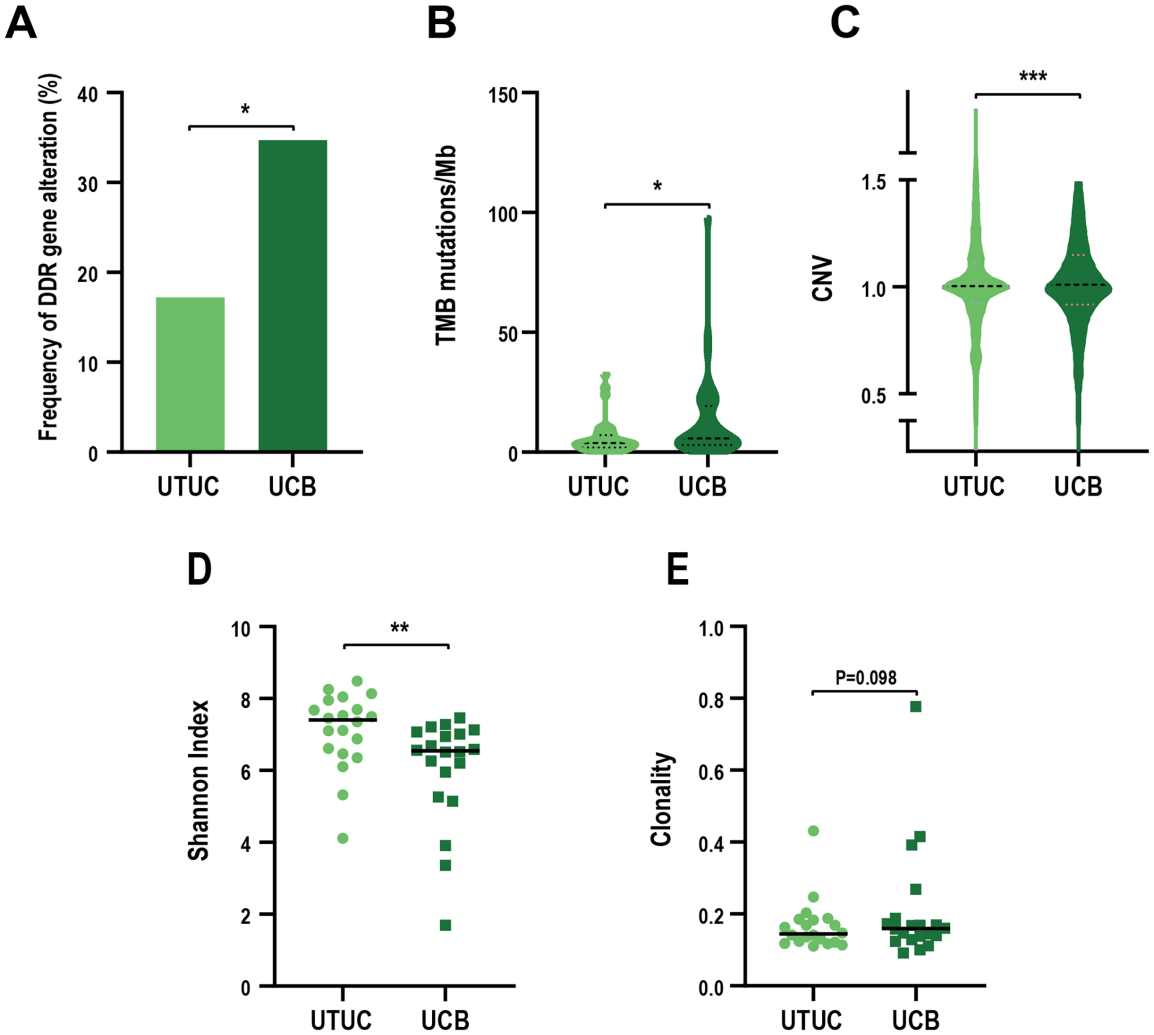
Immunotherapeutic potential of UTUC and UCB. (A–C) Comparison of DDR gene mutations (A), TMB (B), and CNV alteration (C) in UTUC and UCB cohorts. *p < 0.05, ***p < 0.001. (D) Comparison of TCR diversity between paired UTUC and UCB samples. The Shannon index was used to represent TCR diversity. **p < 0.05. (E) Comparison of TCR clonality between paired UCUC and UCB samples. The clonality index was used to represent TCR clonality.

Since UCB is more responsive to ICI than UTUC [9], we examined whether pathways with different mutations were responsible for the differences in ICI response. Compared to UTUC, UCB had more deleterious mutations in DDR (Figure 6A) and higher levels of tumor mutation burden (TMB) (Figure 6B) and CNVs (Figure 6C). In addition, deleterious mutations in DDR were highly associated with the level of TMB (Figure S6A). TCR sequencing of the UTUC and UCB samples indicated that T‐cell clonal diversity was higher in UTUC than UCB (Figure 6D) even though they shared a clonal origin (Figure S6B). Furthermore, UCB tumors have a higher degree of T‐cell clone, which further indicates that UCB might respond better to immunotherapy (Figure 6E). Previous studies showed that T‐cell density and PD‐L1 expression were closely correlated with the response to immunotherapy [41, 42]. Therefore, we compared the T‐cell density and PD‐L1 expression between UTUC and paired UCB samples from the 20 patients. Our results showed that the densities of CD3^+^, CD4^+^, or CD8^+^ T cell were comparable in UTUC and paired UCB samples (Figure S6C,E). The PD‐L1 is undetectable in 16 UTUC samples and their paired UCB samples. PD‐L1 is found in both UTUC and UCB samples of four patients. PD‐L1 expression (TPS score or CPS score) is less than 5% (Figure S6F), indicating that the expression of PD‐L1 in the paired UTUC and UCB was consistent. Altogether, these findings suggest that mutations in distinct pathways and the diversity of T‐cell clones might affect UCB responsiveness to ICI.

## 4 Discussion

Although UTUC and UCB are similar histomorphologically and therapeutically, they are distinct epidemiologically and clinicopathologically. We found that chromatin regulatory genes are frequently mutated in both UTUC and UCB, and therefore this pathway likely plays important role in UC development and progression. Since mutations in *KMT2C, SMARCA4*, and *EP300* led to rapid relapse and progression of NMIBC, more attention to monitoring the recurrent tumors and more aggressive treatment regimen should be applied to the recurred tumors. We have also found that GATA3 and CK5 double‐positive patients in our cohort are more frequent than usual. Patients in this subgroup were found with higher mutation frequencies in *SMARCA4, ARID1A*, and *EP300* than those with luminal subtype, and have a better prognosis than those with basal subtype. Notably, the UTUC and UCB tumors in the same patient could be either clonally related or independent albeit mutated chromatin pathway genes are enriched in the shared clones. Finally, different mutation frequencies in DDR pathway, TMB, CNV, and TCR might be the contributing factors for the distinct responses to ICI between UTUC and UCB.

Chromatin plays a central role in transcriptional regulation and the maintenance of genomic stability, and mutations in chromatin‐regulatory genes are important contributing factors in cancer initiation and progression [43]. Based on the 2017 Cancer Genome Atlas (TCGA) MIBC cohort, chromatin regulatory genes are the second most frequently mutated genes after the TP53/RB tumor suppressor pathway [18]. UTUC tumors also showed a high frequency of mutations in chromatin regulatory genes [15]. Here, we focused on genetic alterations of chromatin regulatory genes with a high frequency of mutations including *KDM6A*, *KMT2D*, *KMT2C*, *ARID1A*, *CREBBP*, *EP300*, and *SMARCA4* as described previously [15]. We found that the chromatin pathway genes are the most frequently mutated in both UTUC and UCB. Although alterations of the chromatin pathway were not associated with the stage, grade, progression, or molecular subtypes of either tumor type, we found that alterations of *KMT2C*, *SMARCA4*, or *EP300* lead to rapid relapse and progression of NMIBC. Previous research have linked these genes with different cancers. Deficient homologous recombination‐mediated double‐strand break DNA repair due to low *KMT2C* activity resulted in endogenous DNA damage and genomic instability [44]. Alterations of *SMARCA4*, a member of the SWI/SNF chromatin remodeling complex, are associated with cell differentiation [45]. *EP300* mutations are associated with increased TMB and promote antitumor immunity [46]. The roles of these factors in UC recurrence and progression warrant further investigation.

The 2‐year bladder cancer recurrence rate after radical nephroureterectomy is 22%–47%, and the incidence of subsequent UTUC is 2.6% and 3%–5% in NMIBC and MIBC patients, respectively [7, 47]. About 17% of the patients were diagnosed with both UTUC and UCB simultaneously [48]. There is some debate whether UTUC and UCB tumors in the same patient are clonally related recurrences or represent distinct primary tumors. Two studies have shown that 100% (29/29) and 73.3% (11/15) of recurrent UCB after primary UTUC had the same clonal origin [15, 49]. A meta‐analysis found that more than 90% of primary UTUC with subsequent UCB were clonally related, and more than 80% of primary UCB with subsequent UTUC were clonally related [23]. Du et al. showed both monoclonal and multiclonal origin patterns in the same patient in five tumor samples [50]. We found about 55% of the samples in our cohort share a clonal origin indicating that UTUC and UCB in the same patient can develop either clonally or independently, and, the clonal relatedness does not depend on which occurs first. Nevertheless, the mutated chromatin pathway genes were enriched in both clonal UTUC and UCB.

Multiple lines of evidence showed that the response rate to ICI treatment of UTUC patients is significantly lower than that of UCB patients [9]. The results from the IMvigor210 Phase II and IMvigor211 Phase III clinical trials showed that atezolizumab conferred higher ORR in UCB patients than in UTUC patients (23% vs. 13%, 18% vs. 11%, respectively). Another two cohorts from the KEYNOTE052 Phase II trial of pembrolizumab and the JAVELIN Phase I trial of avelumab also showed the ORR in UTUC patients is lower (22% vs. 28%, 11% vs. 18%, respectively). DDR mutations, TMB, and CNV have been suggested as contributing factors to ICI efficiency [28, 51, 52]. MIBC with DDR mutations are immunologically hot and responsive to ICI [53]. PD‐L1, associated with TMB, is considered an effective marker for ICI selection [54]. Combination of TMB and CNV stratified prognostic and predictive responses to immunotherapy across metastatic cancers [51]. We found that UCB patients carry higher frequencies of DDR mutations, high TMB, and CNV than UTUC patients. In addition, TCR diversity could be used to predict the response to immunotherapy [55, 56] and Choudhury et al. found that low TCR β‐chain diversity was associated with longer recurrence‐free survival in MIBC and might be optimal candidates for immunotherapy [57]. To our knowledge, we are the first to investigate the TCR in paired UTUC and UCB tumors from the same patient and found that TCR diversity is higher in UTUC even in patients with tumors of the same clonal origin. On the other hand, the T‐cell clonality in UTUC is lower than that of UCB. Therefore, combined with the findings that DDR, TMB, and CNV were more altered in UCB than UTUC, our results support the notion that UCB patients have a better response to ICI than UTUC patients.

One of the limitations of this study is the relatively small sample size. Therefore, studies of large cohorts would be of great significance to verify our results. Another limitation is that different platforms for sequencing were used in our study. However, the majority of the genetic alterations identified by WES could be identified using our gene panels, and the genes, especially chromatin regulatory genes that we focused on in this study, were all included in the panels. Finally, differences in gene expression might also be important for UTUC and UCB, which could not be addressed by our DNA sequencing approach. Further investigation integrating genome, transcriptome, and proteomic analyses would provide further insights into factors explaining the differences between UTUC and UBC.

## Author Contributions

**Shuo Wang:** data curation (equal), formal analysis (equal), investigation (equal), methodology (equal), resources (equal), software (equal), visualization (equal), writing – original draft (equal), writing – review and editing (equal). **Xuzhi Yan:** data curation (equal), formal analysis (equal), investigation (equal), methodology (equal), resources (equal), software (equal), visualization (equal), writing – original draft (equal), writing – review and editing (equal). **Weihua Lan:** data curation (equal), formal analysis (equal), funding acquisition (equal), investigation (equal), methodology (equal), resources (equal), software (equal), visualization (equal), writing – original draft (equal), writing – review and editing (equal). **Yapeng Wang:** resources (equal). **Ze Wang:** resources (equal). **Dali Tong:** resources (equal). **Yao Zhang:** resources (equal). **Qiang Ran:** resources (equal). **Haoyang Li:** resources (equal). **Junhao Jin:** resources (equal). **Haiyang Xiao:** resources (equal). **Jing Xu:** investigation (equal). **Qian Yan:** investigation (equal). **Dianzheng Zhang:** writing – original draft (equal). **Qiang Ma:** investigation (equal). **Hualiang Xiao:** investigation (equal). **Jun Qin:** formal analysis (equal). **Luofu Wang:** conceptualization (equal), data curation (equal), formal analysis (equal), funding acquisition (equal), project administration (equal), supervision (equal), validation (equal), writing – original draft (equal), writing – review and editing (equal). **Jun Jiang:** conceptualization (equal), data curation (equal), formal analysis (equal), funding acquisition (equal), investigation (equal), methodology (equal), project administration (equal), resources (equal), supervision (equal), validation (equal), visualization (equal), writing – original draft (equal), writing – review and editing (equal). **Qiuli Liu:** conceptualization (lead), data curation (lead), formal analysis (lead), funding acquisition (equal), investigation (lead), methodology (lead), project administration (lead), supervision (equal), validation (equal), visualization (equal), writing – original draft (equal), writing – review and editing (equal).

## Ethics Statement

This study was approved by the Research Ethics Committee of Daping Hospital, Army Medical University (No: 201896), and written informed consent from each patient.

## Conflicts of Interest

The authors declare no conflicts of interest.

## Supporting information


**Figure S1.** Differences in genetic alterations between UTUC and UCB. (A) Significant differences in the frequency of genomic mutation of the top mutated genes between TCGA UTUC and our UTUC cohort. (B) Significant differences in the frequency of genomic mutations for the top mutated genes among TCGA UCB cohort and our UCB cohort. (C and D) The mutation types for chromatin regulatory genes in UTUC (C) and UCB (D). (E) Comparison of the mutation frequencies in the top mutant genes in UTUC and UCB. (F) The mutation frequencies of ZNF668 in UTUC and UCB.

**Figure S2.** Genetic alterations associated with tumor stage, grade, and prognosis. (A and B) The associations of genetic alterations in the TP53/MDM2 pathway with tumor T‐stage (A) and grade (B). (C) The associations of genetic alterations in the RTK/RAS pathway with tumor grade. (D) Representative image of Ki67 immunohistochemical staining. The expression of Ki67 ≤ 25% was considered low, and the expression of Ki67 > 25% was considered high. (E and F) Kaplan–Meier analysis showing the associations of Ki67 expression with NMIBC recurrence (E) and progression (F).

**Figure S3.** Mutational signature analysis. (A and B) The proportion of single base substitution patterns in each case of UTUC (A) and UCB (B) cohorts. (C) The association of genetic alterations in RTK/RAS pathway with or without SBS22 (for exposure to aristolochic acid) signature in UTUC patients. (D) The association of genetic alterations in the PI3K/AKT pathway with SBS6 (for defective DNA mismatch repair) signature in UCB patients.

**Figure S4.** The molecular subtypes of the patients. (A) The molecular subtypes in the UTUC and UCB cohort with different tumor stages. (B) The molecular subtypes of the 78 MIBC patients in the tissue microarrays from another cohort.

**Figure S5.** Clonal evolution analysis. The phylogenetic tree shows the evolutionary pattern of paired UTUC and UCB tumors that were thought to have clonal relatedness.

**Figure S6.** Immunotherapeutic potential of UTUC and UCB. (A) TMB analysis in patients with and without deleterious DDR gene alterations (A). (B) TCR diversity in paired UTUC and UCB samples from the 11 patients with tumors of the same clonal origin. *p < 0.05. (C–E) T‐cell density of CD3 (C), CD4 (D), and CD8 (E) in paired UTUC and UCB samples from the 20 patients. (F) The expression of PD‐L1 in paired UTUC and UCB.

Data S1.

## Data Availability

The data generated during current study are publicly available in the GSA database (HRA004401) at https://ngdc.cncb.ac.cn/gsa.
