# Supplementary figures and images for: Genetic Alterations in Chromatin Regulatory Genes in Upper Tract Urothelial Carcinoma and Urothelial Bladder Cancer
Source: Cancer Med. 2024 Nov 8;13(21):e70398. doi: 10.1002/cam4.70398 (PMC11544325; doi:10.1002/cam4.70398)

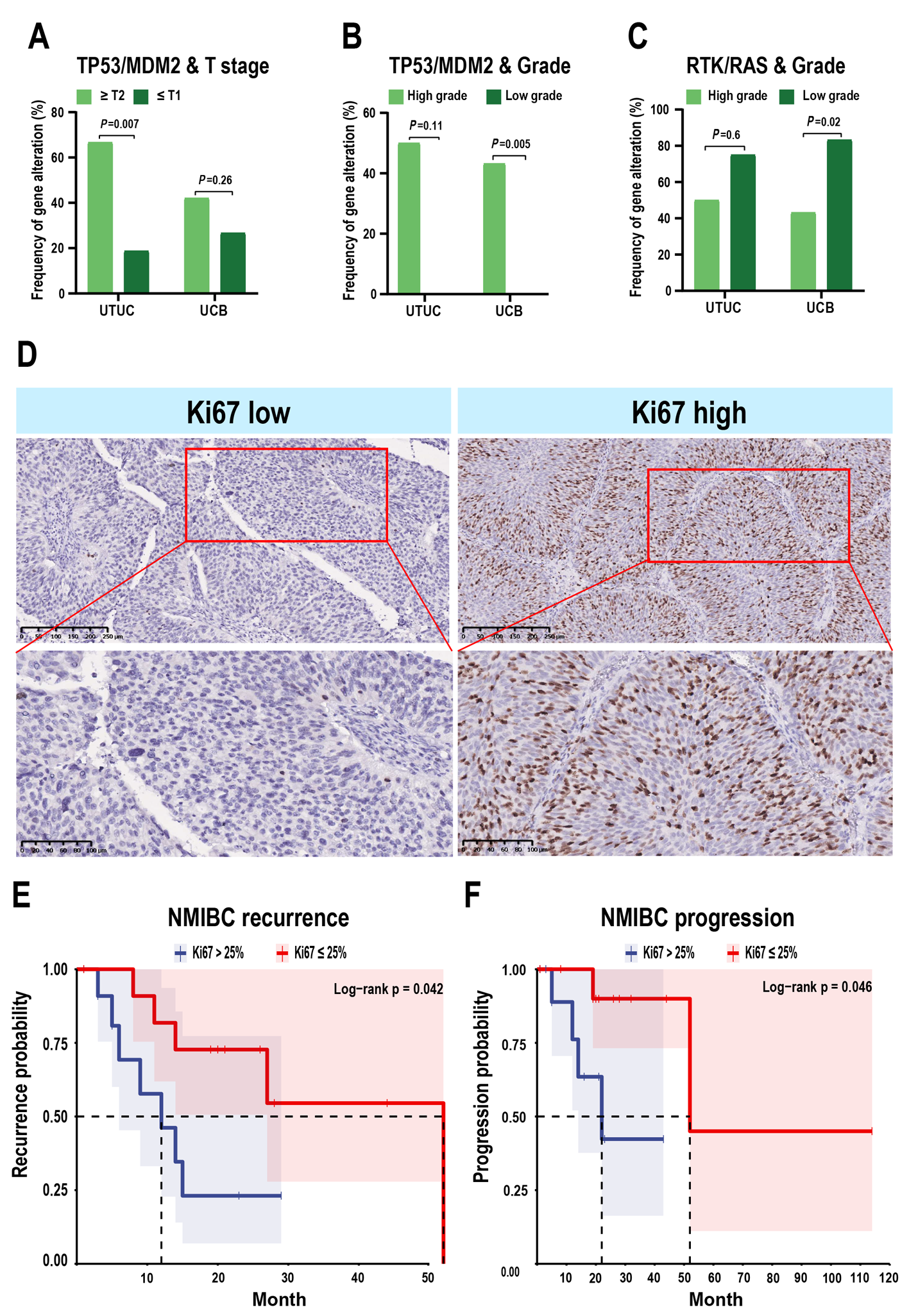

Supplement: Supplementary file 2 — Figure S2. Genetic alterations associated with tumor stage, grade, and prognosis. (A and B) The associations of genetic alterations in the TP53/MDM2 pathway with tumor T‐stage (A) and grade (B). (C) The associations of genetic alterations in the RTK/RAS pathway with tumor grade. (D) Representative image of Ki67 immunohistochemical staining. The expression of Ki67 ≤ 25% was considered low, and the expression of Ki67 > 25% was considered high. (E and F) Kaplan–Meier analysis showing the associations of Ki67 expression with NMIBC recurrence (E) and progression (F). [file CAM4-13-e70398-s007.tif]

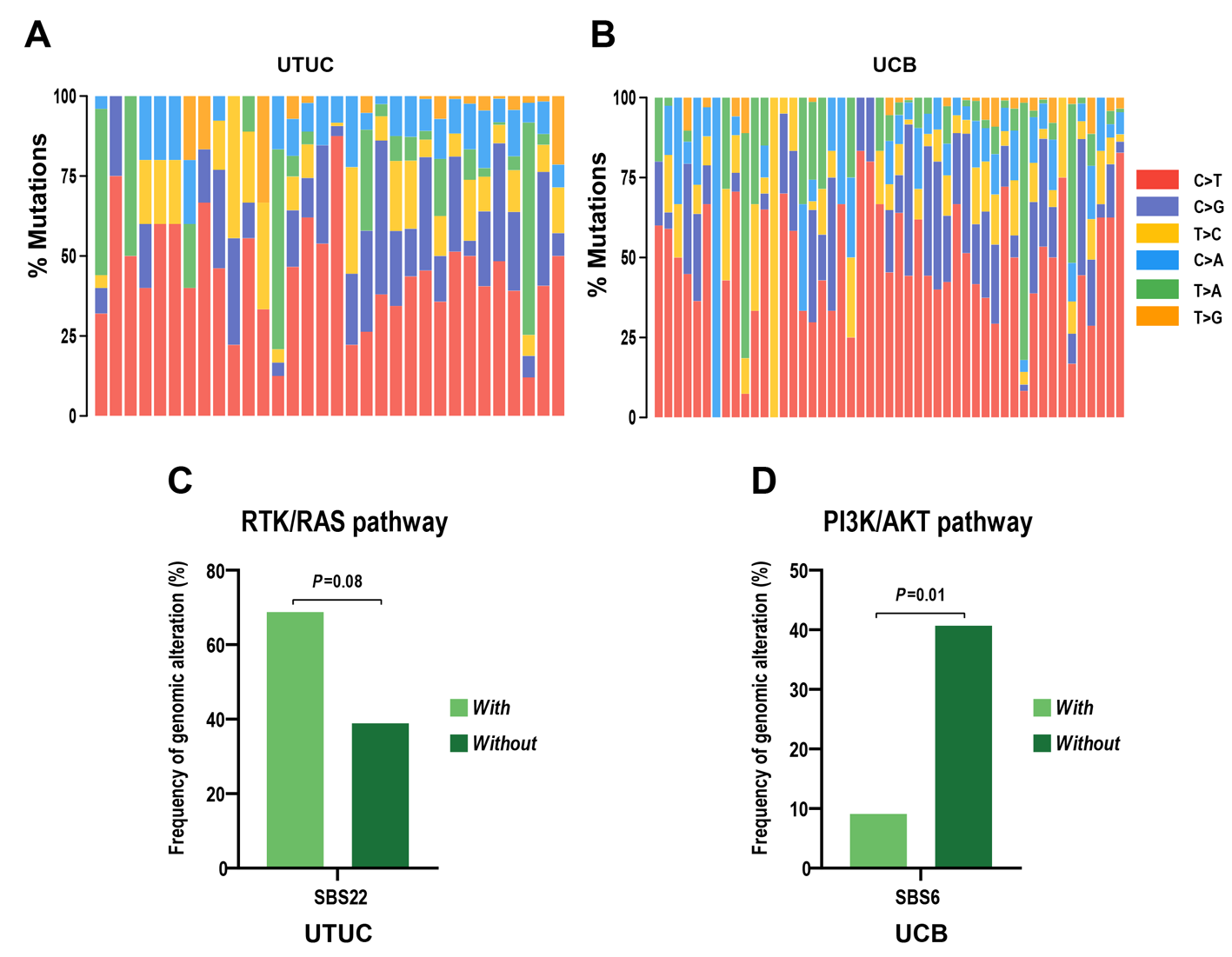

Supplement: Supplementary file 3 — Figure S3. Mutational signature analysis. (A and B) The proportion of single base substitution patterns in each case of UTUC (A) and UCB (B) cohorts. (C) The association of genetic alterations in RTK/RAS pathway with or without SBS22 (for exposure to aristolochic acid) signature in UTUC patients. (D) The association of genetic alterations in the PI3K/AKT pathway with SBS6 (for defective DNA mismatch repair) signature in UCB patients. [file CAM4-13-e70398-s003.tif]

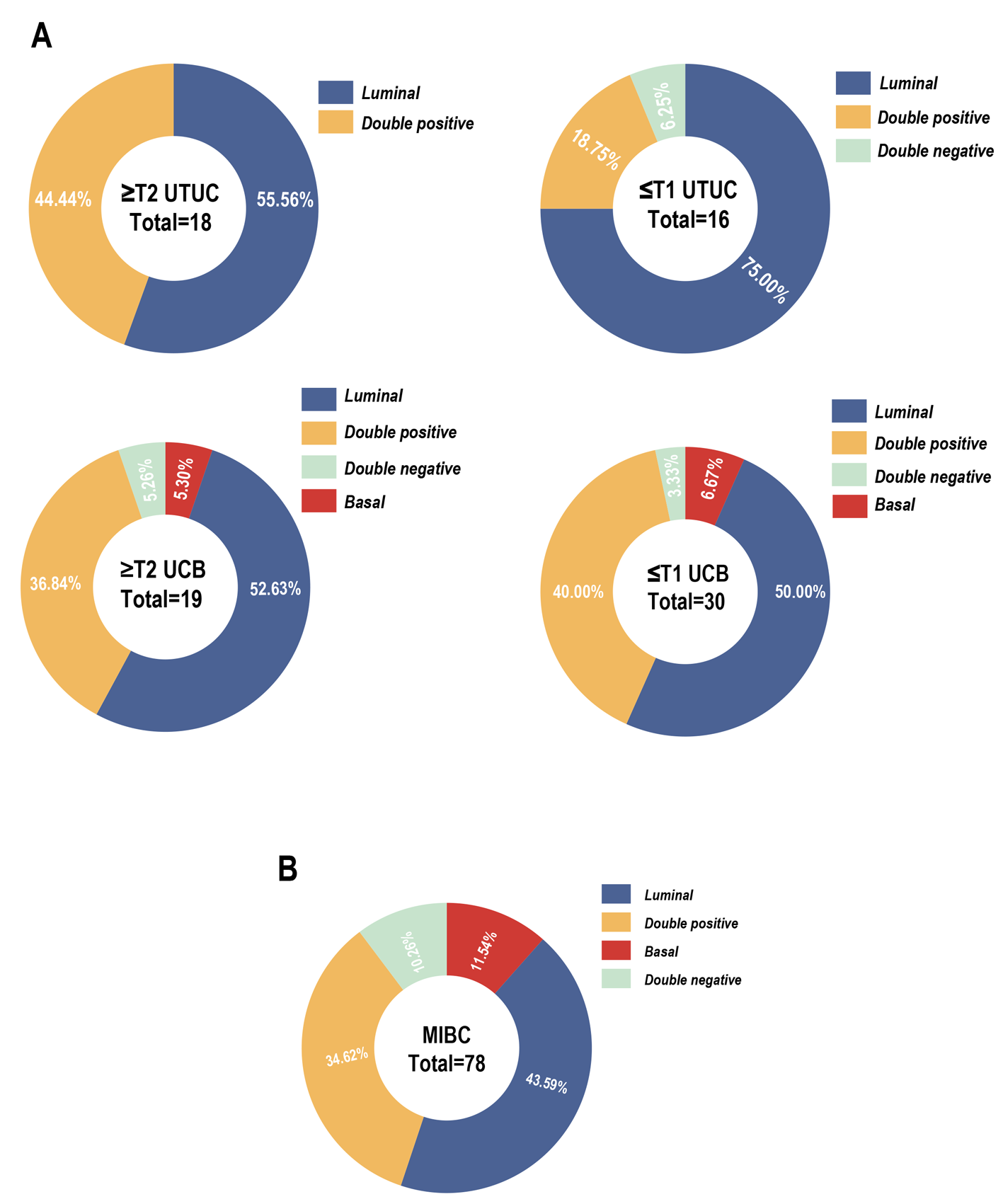

Supplement: Supplementary file 4 — Figure S4. The molecular subtypes of the patients. (A) The molecular subtypes in the UTUC and UCB cohort with different tumor stages. (B) The molecular subtypes of the 78 MIBC patients in the tissue microarrays from another cohort. [file CAM4-13-e70398-s005.tif]

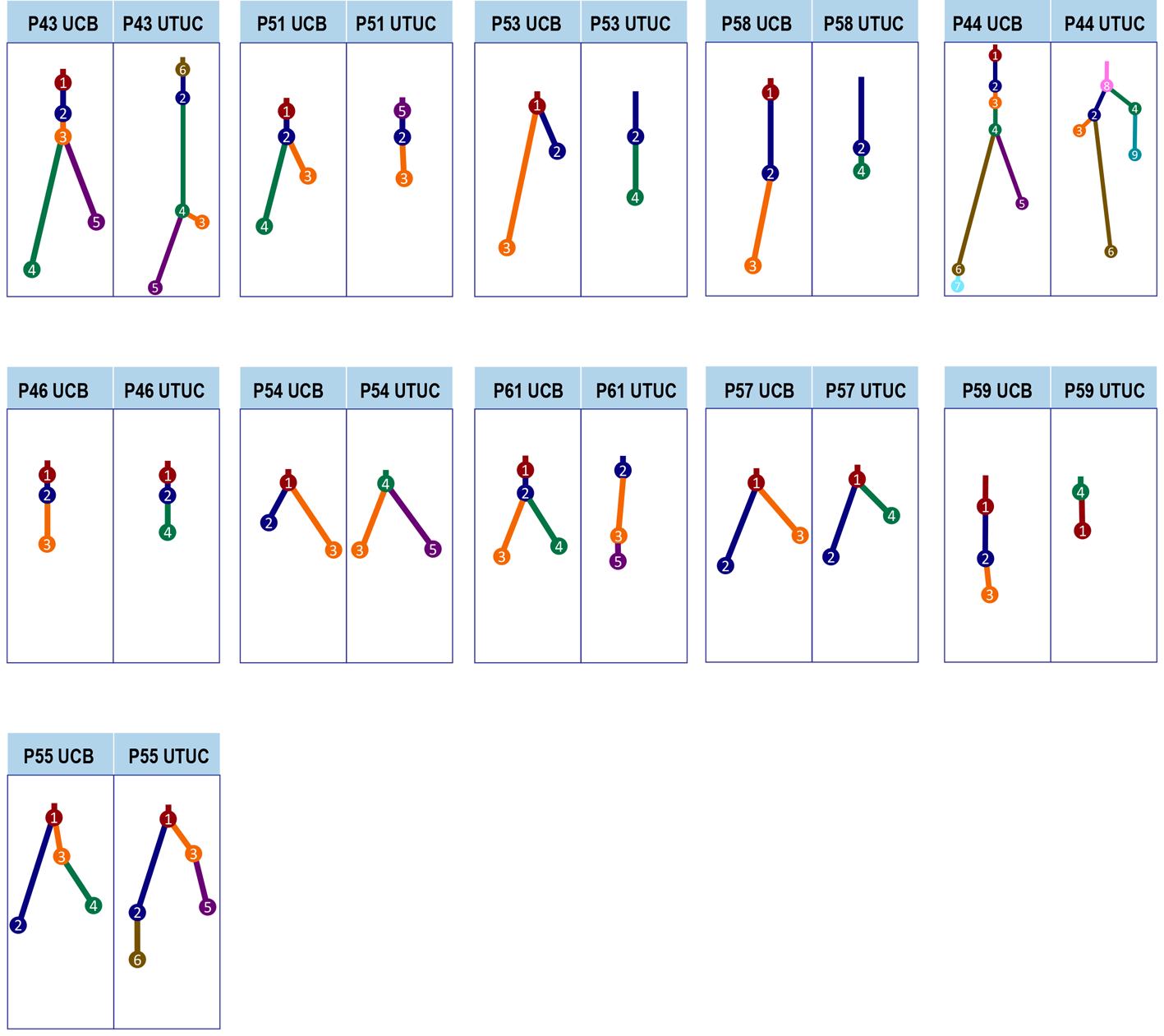

Supplement: Supplementary file 5 — Figure S5. Clonal evolution analysis. The phylogenetic tree shows the evolutionary pattern of paired UTUC and UCB tumors that were thought to have clonal relatedness. [file CAM4-13-e70398-s002.tif]
